# Understanding the Puzzle of Primary Breast Lymphoma: An Experience in a Tertiary Care Center in North India

**DOI:** 10.7759/cureus.68152

**Published:** 2024-08-29

**Authors:** Kusum Yadav, Nidhi Gupta, Akanksha Sharma, Manish Kumar

**Affiliations:** 1 Pathology, Dr. Ram Manohar Lohia Institute of Medical Sciences, Lucknow, IND; 2 Pathology, Dr. KNS Memorial Institute of Medical Sciences, Lucknow, IND; 3 Pathology, Rama Medical College and Research Hospital, Hapur, IND

**Keywords:** cd30, alcl in breast, dlbcl in breast, breast malignancy, primary breast lymphoma

## Abstract

Background

Breast carcinoma is the most common cause of death in women arising primarily from either epithelial or stromal components. Primary breast lymphoma (PBL) is a less common type of neoplasm arising from the lymphoid tissue and is classified as non-Hodgkin’s lymphoma (NHL). PBL is a rare tumor that accounts for less than 1% of total primary malignant neoplasms of the breast. The majority of PBLs are of B-cell origin; the most common histological type is diffuse large B-cell lymphoma (DLBCL) followed by follicular lymphoma (FL) and other T-cell and B-cell lymphomas.

Material and methods

We conducted this five-year retrospective descriptive PBL study from July 2019 to May 2024 at a tertiary care center in North India. We included 11 cases of diagnosed PBL based on morphology and confirmation by at least one lymphoid differentiation marker, that is, leukocyte common antigen (LCA)*. *We retrieved paraffin-embedded blocks from the archive and conducted routine hematoxylin and eosin (H&E) staining and Leishman’s stain. We applied an extended immunohistochemistry (IHC) panel to confirm the microscopic findings*.* We used the Dako^®^ (Agilent Diagnostics & Genomics Group) antibody with a dilution of 1:100.

Results

In this study, patients’ mean age was 46.8 years (32 to 84 years). Radiological findings indicated that the tumor was indistinguishable from carcinoma. Microscopic findings showed that cells were medium to large lymphoid cells displaying moderate pleomorphism, centrally placed nuclei, vesicular to coarsely clumped chromatin, and scant cytoplasm. IHC showed one case of T-cell origin (ALCL) and 10 of B-cell origin. Among the B-cell cases, four were DLBCL, four were high-grade NHL likely DLBCL and two were high-grade lymphoma.

Conclusion

Primary breast lymphoma is a rare tumor that closely resembles breast carcinoma clinically as well as radiologically. This study is an effort to highlight the importance of clinical, radiological, and histopathological parameters as well as immunohistochemical features in confirming diagnosis. We also emphasize a treatment protocol and survival outcome in hopes that this knowledge will provide an understanding of early diagnosis and proper management of disease.

## Introduction

Breast carcinoma is the most common cause of death in women, arising primarily from either epithelial or stromal components [1]. Primary breast lymphoma (PBL) is a less common type of neoplasm arising from the lymphoid tissue and is classified as non-Hodgkin’s lymphoma (NHL). It is a rare tumor that accounts for less than 1% of total primary malignant neoplasms of the breast [2,3]. The earliest description of PBL occurred in 1959 [2]. The majority of PBLs are of B-cell origin; the most common histological type is diffuse large B-cell lymphoma (DLBCL) followed by follicular lymphoma (FL) and other T-cell and B-cell lymphomas [4]. Classical Hodgkin’s lymphoma and precursor B-cell lymphoma rarely occur in PBL [5]. The predominant type of T-cell origin lymphoma is anaplastic large-cell lymphomas (ALCL) [6]. Different histological variants of PBL have distinct epidemiological features, prognostic parameters, and required treatments [7]. Because of similar looking clinical as well as radiological features of PBL with primary breast carcinoma, doctors often miss early diagnosis, thus delaying a definite diagnosis [7,8]. Researchers have poorly defined PBL in the literature in terms of the smaller number of patients reported, not defining initial clinical characteristics, pathological features as well as prognostic factors. Because a treatment plan for breast carcinoma that includes targeted hormonal therapy is entirely different from a treatment plan for lymphoma, early and definite diagnosis with the help of morphology and IHC plays a pivotal role. Therefore, to improve our understanding of cases of clinical, pathological, radiological, and prognostic features, we conducted a study of 11 cases reported in a tertiary care center in the past five years.

## Materials and methods

We conducted this retrospective cross-sectional study of PBL (from July 2019 to May 2024) with cases diagnosed in the Department of Pathology, Dr. Ram Manohar Lohia Institute of Medical Sciences, a tertiary care center in North India. The aim of the present study was to highlight the clinical characteristics and disease progression, investigate the histological and immunohistochemical features, and record the survival outcome of primary PBL cases with the current treatment plan. We included 11 cases of diagnosed PBL based on morphology and confirmation by at least one lymphoid differentiation marker, that is, leukocyte common antigen (LCA). We excluded cases negative for LCA (negative membranous staining) and those immunoreactive for any other differentiation markers such as epithelial (CK), melanocytic (HMB45, MELAN A), and mesenchymal (vimentin, desmin) from the study. Since we have conducted a retrospective study, all demographic, clinical, treatment & survival data were obtained from the archive or medical record department. Treatment and follow-up data were obtained in collaboration with the Department of Clinical Hematology (Table 1).

**Table 1:** Clinical and demographic characteristics of cases enrolled ^%^MRM- Modified radical mastectomy; *Rapid progression of disease was considered when the clinical presentation of symptoms was over weeks or months; ^&^Slow progression of disease was considered when the disease was over months or years. Lt. - Left, R. - Right.

Case No.	Age (Yrs)/Sex (M/F)	Site	Specimen received	Tumour size clinically (cm)	Presenting symptoms	Progression	Distant metastasis
1	29/F	Rt. Breast	Blocks for review	-	Breast lump	^*^Slow	No
2	35/F	Lt. Breast	Core biopsy	2.5x2.0x1.0	Breast lump with discharge	^&^Rapid	No
3	50/M	Rt. Breast	Core biopsy	1.5x1x0.5	Breast lump	Slow	No
4	41/F	Lt. Breast	Core biopsy	2.5X2X0.5	Breast lump	Slow	No
5	53/F	Rt. Breast	Core biopsy	1.5x1x0.5	Breast lump	Slow	No
6	50y/F	Rt. Breast Lymph node	Blocks for review	-	Breast lump	-	No
7	84/F	Lt. Breast	Core biopsy	1.5x1.0x0.5	Lt. Breast	Rapid	No
8	32/F	Rt. Breast with axillary lymph nodes	^%^MRM specimen	15x12.5x6.9	Breast lump followed by axillary lump	Rapid	Yes
9	42/F	Lt. Breast	Core biopsy	1x1x0.5	Breast lump	Slow	No
10	56/F	Lt. Breast	Core biopsy	1.2x1.0x0.8	Breast lump	Slow	No
11	52/F	Rt. Breast	Core biopsy	1.5x1.2x0.8	Breast lump	Slow	No

We retrieved paraffin-embedded blocks from the archive and conducted routine H&E staining, Leishman’s stain and Periodic Acid Schiff (PAS) stains. We applied an extended IHC panel to confirm the microscopic findings (Table 2).

**Table 2:** Histological type, immunohistochemical analysis and survival outcome of cases IHC - Immunohistochemistry; ALCL - Anaplastic large B-cell lymphoma; DLBCL - Diffuse large B-cell lymphoma; R-CHOP - Rituximab, cyclophosphamide, doxorubicin, oncovin, and prednisolone; CHOP - Cyclophosphamide, hydroxydaunorubicin, oncovin, and prednisone.

Case NO.	Histological type	Double/Triple Expressor	IHC (Positive)	IHC (Negative)	Chemotherapy Regime	Survival Outcome
1	ALCL	-	LCA, CD3, CD30	CK, CD20, c MYC, ALK MUM-1, BCL2	CHOP	Taken x 3 cycles, plan for 6 cycles
2	DLBCL-Non-Germinal center type-)	Double expressor	LCA, CD3, BCL2 MUM-1, c MYC Ki 67(98 to 100%)	CK, CD20, CD30,CD10	R-CHOP	Taken x 9 cycles
3	DLBCL	Double expressor	LCA, CD20, BCL2 MUM-1, c-MYC, Ki-67(>90%)	CK, CD3 CD30	R-CHOP	Taken x 6 cycles, in follow up
4	DLBCL-Non-Germinal center type	Double expressor	LCA, CD20, BCL2, c-MYC, MUM-1,Ki67(>90%)	CK, CD3 CD30, CD10	R-CHOP	Taken x 6 cycles, Recurrence occurs
5	High-grade NHL-B cell type	-	LCA, CD20 Ki-67(40-50%)	CK, CD3, c MYC, MUM-1, BCL2	R-CHOP	Taken x 6 cycles, in follow up
6	High-grade NHL-Likely DLBCL	-	LCA, CD20 Ki-67(60-70%)	CK, CD3, c MYC, BCL2	R-CHOP	Taken x 6 cycles, in follow up
7	DLBCL Non-Germinal center type	Double expressor	LCA, CD3, CD20, BCL2 MUM-1, c-MYC, Ki-67(>90%)	CK, MUM-1	R-CHOP	The patient died after three months of diagnosis
8	High-grade NHL-Likely DLBCL with nodal metastasis	-	LCA, CD3, CD20, BCL2 Ki-67(>70-80%)	CK, MUM-1, c-MYC,	R-CHOP	Taken x 4 cycles, plan for 6 cycles than radiotherapy
9	High-grade NHL-Likely DLBCL	-	LCA, CD20 Ki-67(50-60%)	CK, MUM-1	-	-
10	High-grade NHL-Likely DLBCL	-	LCA, CD20 Ki-67(50-60%)	CK, CD3, MUM-1, BCL2	R-CHOP	Taken 2 cycles then lost in follow up
11	High-grade NHL-B cell type	-	LCA, CD20 Ki-67(60-70%)	CK, CD3, c MYC, MUM-1, BCL2	R-CHOP	Taken x 6 cycles, in follow up

For IHC staining, 3-5 μ sections were cut and deparaffinization was done with xylene and alcohol. For antigen retrieval, deparaffinized sections were placed in a microwave in tris(hydroxymethyl) aminomethane-ethylenediaminetetraacetic acid (TRIS-EDTA) (TRIS-1.21gm, EDTA 0.37 gm, Tween 20 -500 µl) buffer (pH 9.0)/citrate buffer (pH 6.0) for (98°C for 25 mins) and cooled to room temperature. Sections were treated with Dako® (Agilent Diagnostics and Genomics Group) peroxidase blocking reagent for 10 minutes to block endogenous peroxidase activity, then the primary antibody was added. Excess buffer was wiped off and sections were covered with link antibody (secondary) for 30 minutes at room temperature. Slides were washed two times with TRIS buffer for 5 min each. We used the Dako® antibody with a dilution of 1:100 as a secondary antibody.

## Results

Clinical and demographic findings

Of the 11 cases, 10 were women, and one was a man, with a male-to-female ratio of 0.1:10. The mean age of presentation was 46.8 years, ranging from 32 to 84 years. We found a right breast lump in six cases and a left breast lump in five. One case showed ipsilateral axillary lymph node involvement. In most of the cases, we received a core biopsy specimen (case 8); the remainder were slides and blocks for review (case 2) and modified radical mastectomy (case 1). The most common presentation was a painless breast lump followed by heaviness, occasional skin changes, and discharge (case 2). We mainly found the breast lump to be slowly progressive. However, two cases (cases 2 and 8) showed rapid progression, with one case showing axillary node involvement. We saw metastasis only in one case (case 8, Table 1). 

Radiological findings

**Figure 1:**
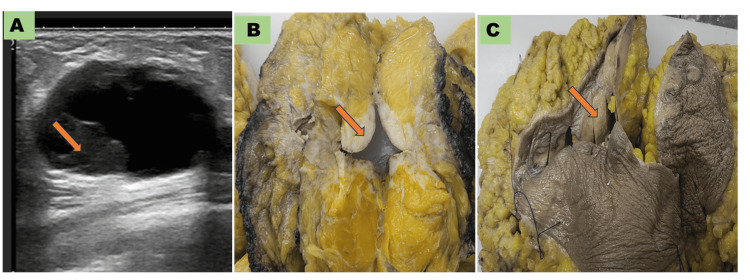
Representative ultrasonography image and gross photographs of cases (A) Ultrasonography image of case 2 displaying mixed solid cystic changes with irregular borders; (B) Gross photomicrograph of Modified Radical Mastectomy breast (case 8) with an arrow showing tumor area; (C) Gross photomicrograph of Modified Radical Mastectomy breast (case 8) with an arrow showing skin nodule infiltrated by tumor cells.

High-resolution ultrasonography of the breast showed nonspecific findings, including hypoechoic to mixed echogenic mass with irregular borders and increased vascularity (Figure 1). These findings were radiologically indistinguishable from breast carcinomas. The majority of cases (10 out of 11) were in categories IV or V of the breast imaging reporting and data system (BI-RADS). Case 2 showed a complex solid cystic lesion with an irregular margin (BI-RADS-IV, Figure 1a).

In one case, positron emission tomography and computed tomography showed a hypermetabolic malignant nodule in the outer upper quadrant of the left breast.

Histopathological findings

**Figure 2:**
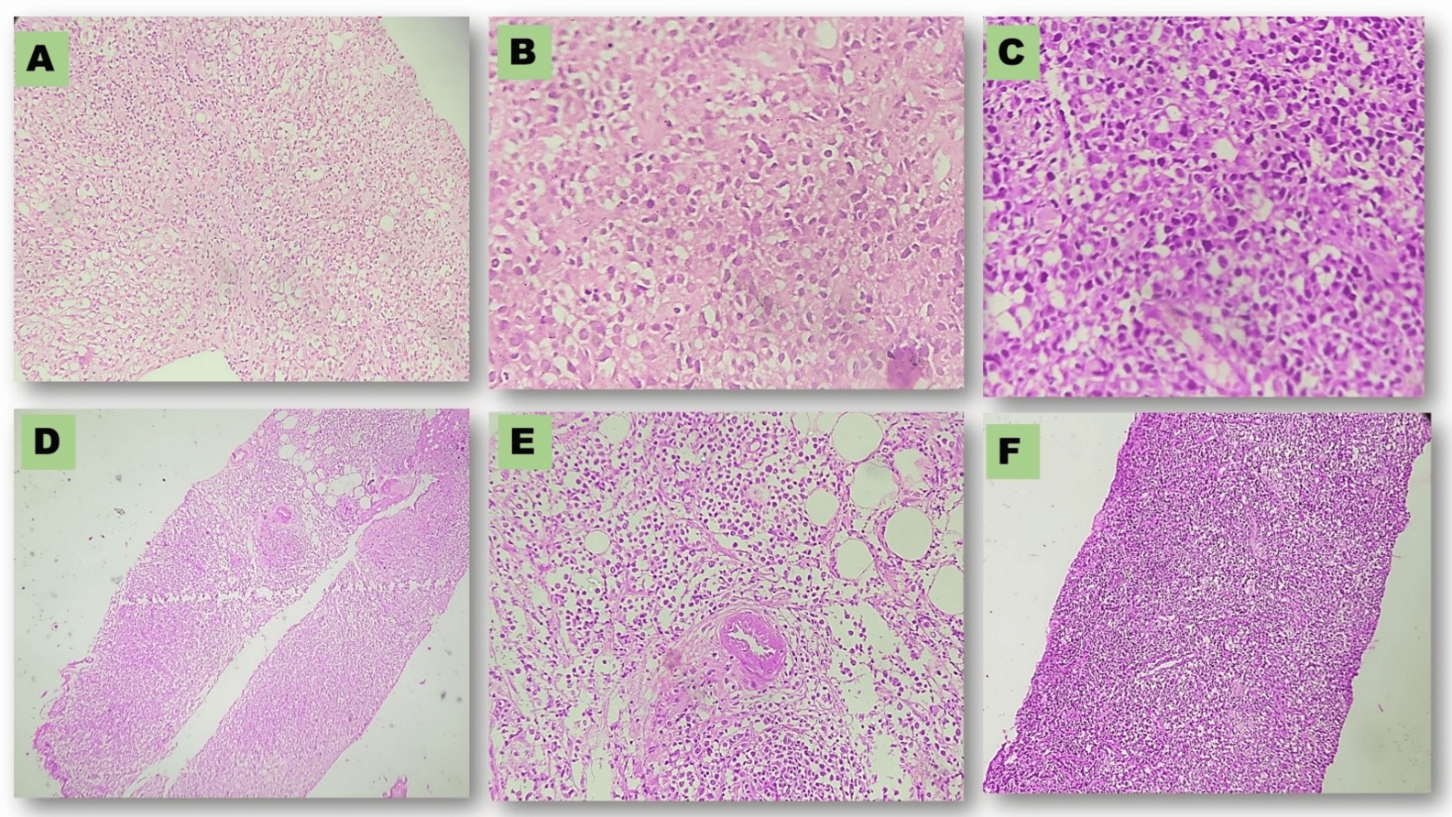
Photomicrographs of histology pictures of cases at various magnifications. (A) Case 1 displaying tumour at 200X; (B) Case 2 displaying tumour at 400X; (C) Case 4 displaying tumour at 400X; (D) Case 5 displaying biopsy at 100X; (E) Case 5 displaying tumour at 400X; (F) Case 6 displaying biopsy at 100X.

Grossly, the tumor was whitish, firm to hard in consistency, and indistinguishable from carcinoma (Figures 1b, 1c). In the majority of cases, histology sections showed multiple small bits of biopsy composed of medium to large lymphoid cells displaying moderate pleomorphism, centrally to eccentrically placed nuclei having vesicular to coarsely clumped chromatin, and scant to moderate amounts of cytoplasm. We observed large, intermixed cells with centrally placed nuclei with prominent nucleoli (cases 2, 3, 4, 7, 8, 9, and 10). We also observed a few bizarre cells (“hallmark” cells) and necrotic bits (case 1). Three cases showed frequent atypical mitosis (cases 2, 3, and 4 in Figure 2).

Common differentials based on morphology were high-grade breast carcinoma, high-grade sarcoma, and lymphoma. 

Immunohistochemical findings

**Figure 3:**
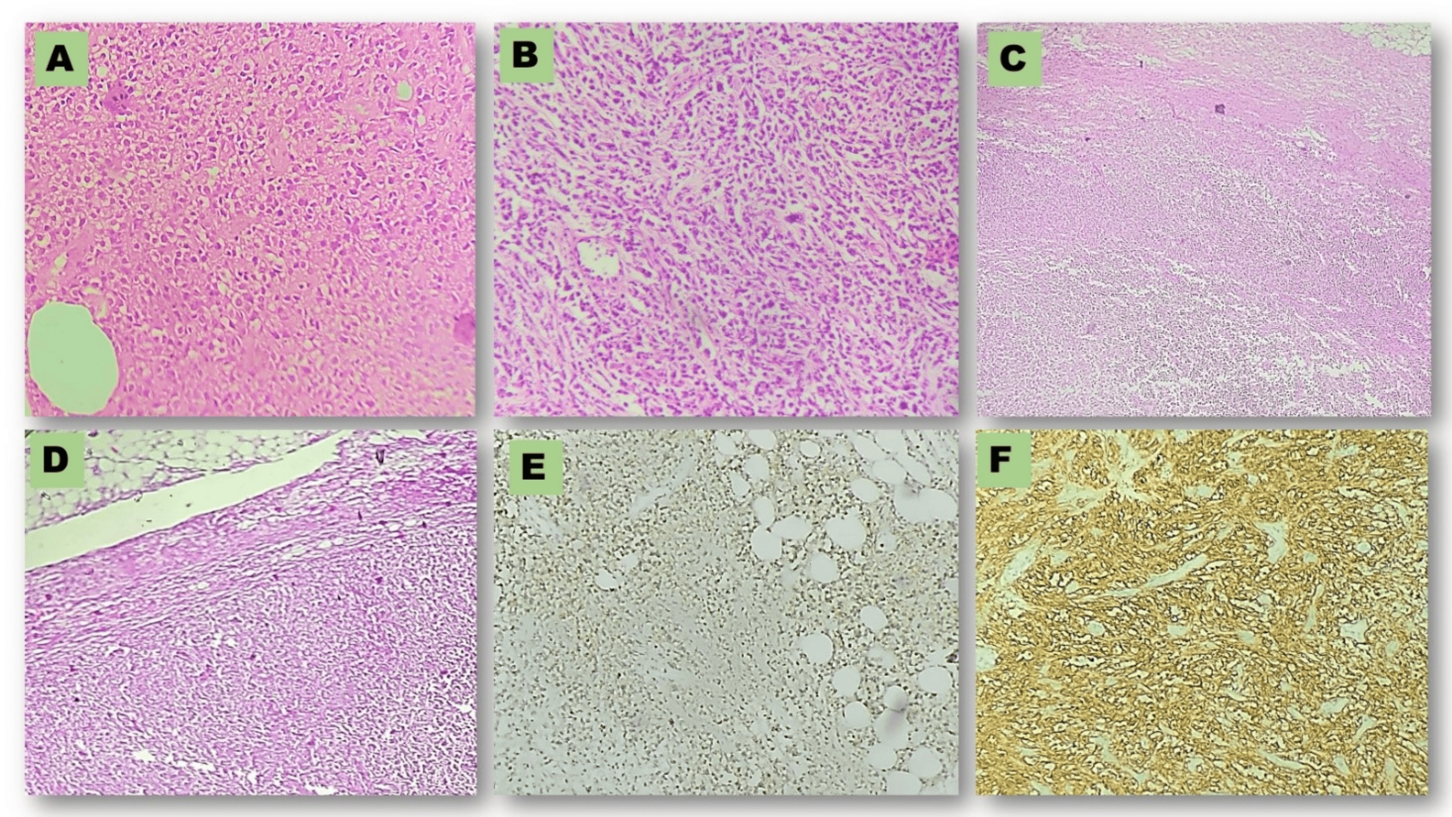
Photomicrographs of histology and immunohistochemistry pictures of cases at various magnifications levels (A) Case 7 displaying tumour at 200X; (B) Case 8 displaying tumour at 400X; (C) Case 8 displaying positive lymph node at 100X; (D) Case 5 displaying positive lymph node at 200X; (E) Positive LCA in case 1; (F) Positive CD20 in case 3.

We considered IHC the primary ancillary technique to rule out morphology-based differentials and to confirm our diagnosis. We considered LCA as the primary lymphoid marker to differentiate between epithelial and mesenchymal malignancies (Figure 3e).

**Figure 4:**
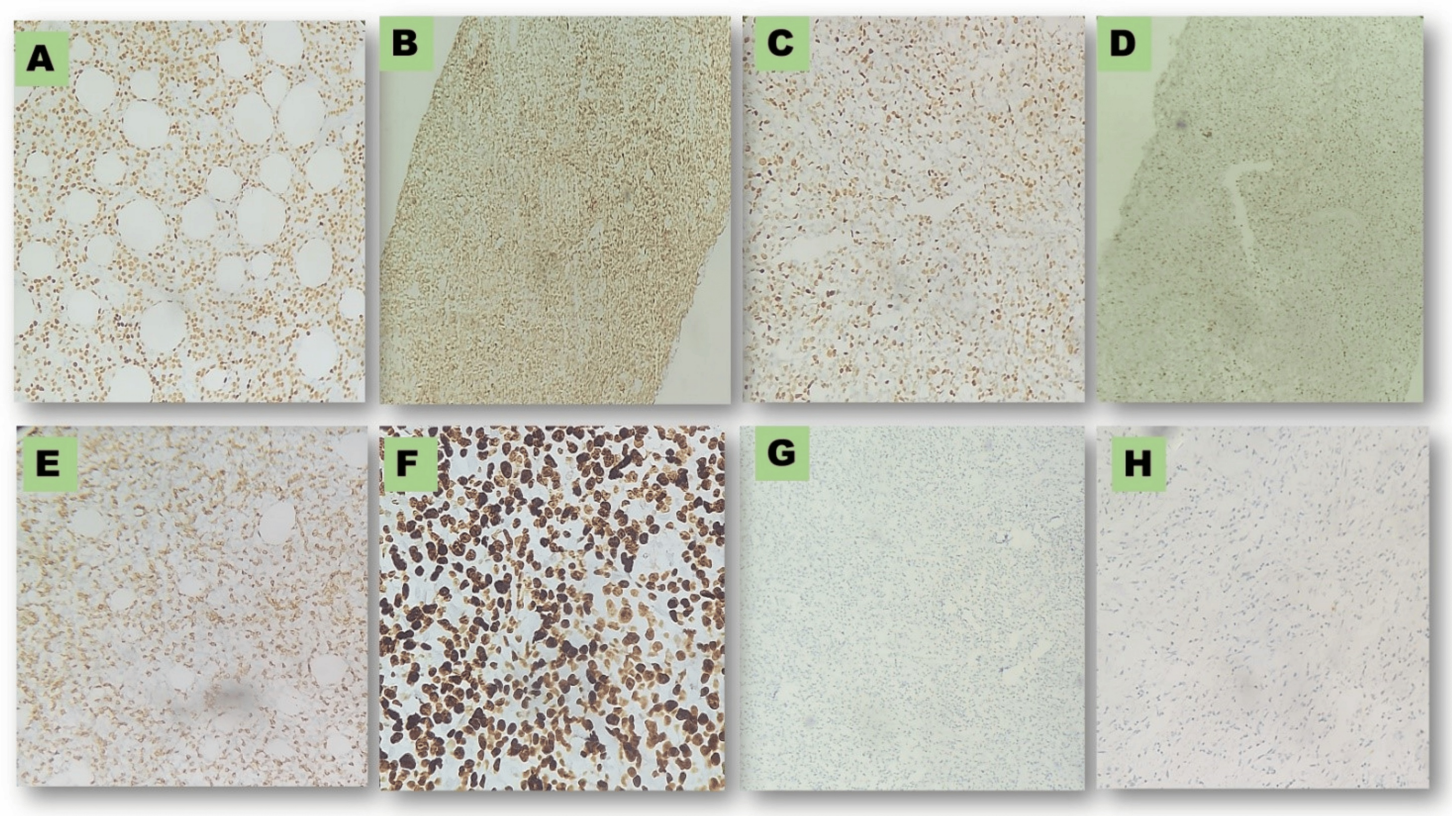
Photomicrographs of immunohistochemistry pictures of cases (A) Positive PAX 5 in case 2; (B) Positive BCL 2 in case 4; (C) Photomicrograph positive MUM-1 in case 4; (D) Positive c MYC in case 4; (E) Positive CD 3 in case 4; (F) ~ 100 % Ki -67 in case 5; (G) Photomicrograph of negative ALK in case 1; (H) Negative CK in case 6.

We used CK to highlight the epithelial component of breast parenchyma. All 11 cases showed bright positivity for LCA and negative immunoreactivity for pan CK. Once we confirmed hematolymphoid malignancy, we applied CD3 (T-cell) and CD20 (B-cell) to confirm the origin of the tumor cells. Of the 11 cases, only one was of T-cell origin; the remaining 10 were of B-cell origin. T-cell NHL tumor cells expressed CD3 and CD30. Therefore, based on morphology and IHC, we made a final diagnosis of ALCL (case 1). Of the 10 B-cell cases, four were DLBCL, four were high-grade NHL likely DLBCL, and two were high-grade lymphoma (Table 2, Figure 4).

Because prognosis of high-grade lymphoma depends upon various factors, we applied IHC markers BCL-2, MUM-1, and C-MYC to look for double hit and triple hit expressor lymphomas. Based on IHC findings in our study, only four cases were double hit lymphoma expressing BCL-2 with MUM-1/C-MYC positivity. No triple negative case was diagnosed. Antigen Kiel 67 (Ki-67) is a proliferation marker representing how aggressively a tumor behaves. In our study, the mean Ki-67 value was ~57% (nuclear positivity of tumor cells on IHC) varying from near 100 % (case 1) to 40% (case 5).

## Discussion

PBL is a rare tumor occurring in the breast and thus requires extensive workup to investigate. Our study comprised the detailed presentation of 11 primary breast lymphomas diagnosed in a tertiary care center in North India. We conducted clinical, radiological, histopathological, and IHC examinations along with treatment and survival scenarios. Inclusion criterion of cases was histomorphology displaying infiltration by atypical lymphoid cells, which we further confirmed by positivity of LCA. We applied CK to rule out the main differential diagnosis of breast carcinoma and to highlight intrinsic breast parenchyma. We applied CD20 and CD3 to confirm the lineage of lymphoid cells, that is, whether the tumor cells were of T-cell or B-cell origin. In our study, seven out of 11 cases showed CD20 positivity, whereas four showed CD3 positivity. Joks et al. [9] conducted a thorough review of the literature and concluded that up to 50% PBL are of B-cell origin. Sakhri et al. [10] documented 13 cases, of which 10 were LBCL.

In our study, the majority of cases were DLBCL. Researchers consider that the causes of primary breast DLBCL are postmenopausal increase in estrogen levels, various inflammatory and autoimmune diseases, pregnancy, and lactation [11-13]. Researchers have also reported Hodgkin’s lymphoma in the breast [14] and even bilateral breast lymphoma with axillary lymph node involvement [15]. We also investigated one case of PBL with ipsilateral axillary node involvement; all 13 cases were positive for tumor metastasis. The Hans algorithm is a practical approach to classify DLBCL, so we applied an IHC panel according to this algorithm (CD10, BCL6, and MUM-1) and found that four out of seven DLBCL cases were non-germinal centre type. Earlier studies supported our findings [16]. The Ki-67 proliferation index is usually high (~80%) in PBL-DLBCL [17]. Our study also supports the Ki-67 ranges (40%-100%) with a mean value of ~57%. Because PBL-DLBCL is an aggressive malignancy, systemic chemotherapy followed by radiotherapy or immunotherapy is the main treatment protocol. In contrast to mastectomy in breast carcinoma, PBL is associated with a poorer prognosis and a greater chance of recurrence [18]. In our study, nine out of 11 cases received rituximab, cyclophosphamide, doxorubicin, Oncovin®, and prednisolone (R-CHOP) chemotherapy, and one received induction with cyclophosphamide, hydroxydaunorubicin, Oncovin®, and prednisone (CHOP). In primary T-cell breast lymphomas, ALCL is the commonest type of breast lymphoma, followed by peripheral T-cell lymphoma and follicular T-cell lymphoma [19]. Most cases of ALCL are associated with breast implants; researchers have so far reported only ~50 cases of primary isolated ALCL. Morphologically, high-grade ALCL is characterized by large pleomorphic “hallmark” cells with irregular nuclear contours and enlarged and irregular nuclei. Case 1 in our study showed similar morphological pictures.

These tumor cells show immunopositively for CD30 and may show anaplastic lymphoma kinase (ALK) positivity or ALK negativity. In our study, atypical cells were CD30 positive and ALK negative [19,20]. ALK-negative ALCL commonly occurs in older patients. ALK-negative lymphomas have a poorer prognosis and thus require an intensive chemotherapy regime. The most common chemotherapy regime is brentuximab vedotin and cyclophosphamide, doxorubicin, etoposide, and prednisone (CHEP), or CHOP chemotherapy alone [21].

Treatment and survival outcome

Systemic chemotherapy, radiation therapy, and immunotherapy along with debulking surgery remain the mainstay of treatment in various PBLs. Six cycles of induction with R-CHOP and radiation therapy along with intrathecal methotrexate in central nervous system prophylaxis are the standard of care in PBL-DLBCL [14]. In our study, nine out of 11 patients received the R-CHOP induction regimen. In case 1, the patient diagnosed with ALCL received the CHOP induction regimen of three cycles and planned additional cycles. Nine out of 11 patients are alive, one died three months after diagnosis, and one lost in contact during follow-up. Two out of the nine surviving patients are undergoing chemotherapy, and seven have completed six cycles and are in follow-up.

Limitation of study

We conducted our study with only 11 cases diagnosed during the past five years. Such a small sample size is the study’s primary limitation. Our study was limited to a single center, so a multicentric study would be more reliable. One patient was lost in contact during follow-up, this attrition of data could be prevented. Moreover, the patients or their relatives did not secure the digital records of imaging studies, and the physical films were poorly preserved. If we could have prevented this, we could have obtained greater insight about our radiological findings.

## Conclusions

Breast carcinoma poses a significant burden on women’s morbidity and mortality both in the Western world and in developing countries. PBL is a rare tumor that closely mimics carcinoma clinically and in imaging studies. Our case study highlights the clinical, radiological, histopathological, and IHC features along with treatment protocols and survival outcomes. We hope that it will provide insights into early diagnosis and proper management of the disease.
